# T cell activation profiles can distinguish gram negative/positive bacterial sepsis and are associated with ICU discharge

**DOI:** 10.3389/fimmu.2022.1058606

**Published:** 2023-01-10

**Authors:** Canxia Huang, Hui Xiong, Weichao Li, Lu Peng, Yukai Zheng, Wenhua Liao, Minggen Zhou, Ying Xu

**Affiliations:** ^1^ Department of Intensive Care Unit, Sun Yat-sen Memorial Hospital, Sun Yat-sen University, Guangzhou, China; ^2^ Department of Dermatology, Sun Yat-sen Memorial Hospital, Sun Yat-sen University, Guangzhou, China; ^3^ Department of Clinical Laboratory, Sun Yat-sen Memorial Hospital, Sun Yat-sen University, Guangzhou, China; ^4^ Guangdong Provincial Key Laboratory of Malignant Tumor Epigenetics and Gene Regulation, Sun Yat-sen Memorial Hospital, Sun Yat-sen University, Guangzhou, China

**Keywords:** gram negative (G -) bacteria, gram positive (G +) bacteria, sepsis, ICU discharge, CD3 + CD8 + CD69+T cell

## Abstract

**Introduction:**

Sepsis is a life-threatening complication resulting from a dysregulated host response to a serious infection, of which bacteria are the most common cause. A rapid differentiation of the gram negative (G^-^)/gram positive (G^+^) pathogens facilitates antibiotic treatment, which in turn improves patients’ survival.

**Methods:**

We performed a prospective, observational study of adult patients in intensive care unit (ICU) unit and underwent the analysis of peripheral blood lymphocyte subsets, cytokines and other clinical indexes. The enrolled 94 patients were divided into no infection group (n=28) and bacterial sepsis group (n=66), and the latter group was subdivided into G^-^ (n=46) and G^+^ (n=20) sepsis subgroups.

**Results:**

The best immune biomarker which differentiated the diagnosis of G^-^ sepsis from G^+^ sepsis, included activation markers of CD69, human leukocyte antigen DR (HLA-DR) on CD3^+^CD8^+^T subset. The ratio of CD3^+^CD4^+^CD69^+^T/CD3^+^CD8^+^CD69^+^T (odds ratio (OR): 0.078(0.012,0.506), P = 0.008), PCT>0.53 ng/ml (OR: 9.31(1.36,63.58), P = 0.023), and CO_2_CP<26.5 mmol/l (OR: 10.99(1.29, 93.36), P = 0.028) were predictive of G^-^ sepsis (versus G^+^ sepsis), and the area under the curve (AUC) was 0.947. Additionally, the ratio of CD3^+^CD4^+^CD69^+^T/CD3^+^CD8^+^CD69^+^T ≤ 0.2697 was an independent risk factor for poor ICU discharge in G^-^ sepsis patients (HR: 0.34 (0.13, 0.88), P=0.026).

**Conclusion:**

We conclude that enhanced activation of T cells may regulate the excessive inflammatory response of G^-^ bacterial sepsis, and that T cell activation profiles can rapidly distinguish G^-^ sepsis from G^+^ sepsis and are associated with ICU discharge.

## Introduction

Defined as a life-threatening organ dysfunction due to a dysregulated host response to an infection (1), sepsis remains a healthcare problem worldwide (2) with a high mortality rate between one in three and one in six (3–5). Studies indicated that the progressive multiple organ dysfunction syndrome (MODS) is mainly an ominous trajectory from infection to death (6). In addition to the hypoperfusion of the organs/tissues, the immune response inducing a severe macro and microcirculatory dysfunction plays an important role in injuring multiple organs (i.e., the inflammation response induces endothelial dysfunction (7, 8), microthrombus formation (9), and cellular dysfunction (10)). Consequently, dysregulated host immune response is currently accepted as the major cause of MODS.

Bacteria are the most common cause of sepsis (11), and a rapid differentiation of the gram negative (G^-^)/gram positive (G^+^) pathogens facilitates antibiotic treatment, which in turn improves patients’ survival. There are several biomarkers that discriminate the G^-^/G^+^ bacterial sepsis. It is well known (12) that a high procalcitonin (PCT) level is independently associated with G^-^ bacteremia (vs G^+^ bacteremia), however, a meta-analysis of three randomized control trials (RCTs) (13–15) showed that there were no differences in short-term mortality, ICU stay, and length of hospitalization between PCT-guiding protocols antibiotic initiation and usual care. The surviving sepsis campaign (2021) weakly recommended PCT to initiate antimicrobial treatment. Another study (16) demonstrated that a higher interleukin-1 receptor 2 (IL1R2) level could be potentially a new biomarker for G^-^ sepsis versus G^+^ sepsis, while no deep investigation was carried out. To date, few biomarkers have been recommended for differentiation of G^-^/G^+^ bacterial sepsis and improvement of sepsis prognosis effectively.

Researches have suggested that different pathogens in infected patients might lead to different lymphocyte changes (17, 18), which could be closely associated with patients’ prognosis. Several phenotypes of T cells have been proposed regarding development of immune dysfunction in bacterial sepsis. Faulkner et al. (19) found that mortality and release of cytokines in staphylococcal enterotoxin B (SEB)-induced toxic shock model were critically dependent on the presence of TCR αβ T cells in the spleen. Christopher et al. (20) demonstrated that the mucosa-associated invariant T cells (MAIT cells) launched a rapid, robust and distinct hyper-inflammatory response (IFN-γ) to bacterial superantigens, resulting in lethality. In addition, Cheng et al. (18) found that the count of CD4^+^CD28^+^ T cell was significantly lower in carbapenem-resistant enterobacteriaceae (CRE) than non-CRE septic patients, and a lower count of CD4^+^CD28^+^ T cell was significantly associated with a higher mortality. Guignant et al. (21) showed that the increased PD-L1 CD4^+^ T lymphocyte was associated with the elevated occurrence of secondary nosocomial infections and mortality after septic shock. The BIPS study conducted by Velly et al. (22) indicated that the biomarkers mostly facilitating the diagnosis of bacterial infection were consist of myeloid-epithelial-reproductive tyrosine kinase (MerTk) on neutrophils, HLA-DR on monocytes, and plasma metaloproteinase-8 (MMP8). In summary, few studies have concentrated on the effects of comprehensive T lymphocyte subsets on the diagnosis of G^-^ sepsis (versus G^+^ sepsis) and their prognostic value for septic patients.

In this study, we investigated the effects of changes of T lymphocyte subsets (including the active and suppressive biomarkers on CD4^+^/CD8^+^ T-cells), cytokines, and other clinical biomarkers on the diagnosis of patients with bacterial sepsis, and evaluated their relationship with the prognosis of bacterial sepsis.

## Materials and methods

### Patients

Adult critically ill patients admitted to intensive care unit (ICU) of Sun Yat-sen Memorial Hospital Affiliated to Sun Yat-sen University (Guangzhou, China) from March 2020 to July 2022 were enrolled. The inclusion criteria were as follows: (1) age >18 years old, (2) patients who underwent the peripheral blood lymphocyte subset analysis, (3) critically ill patients. The exclusion criteria were as follows: (1) age <18 years old, (2) patient died within 48 h, (3) any condition causing primary or acquired immunodeficiency, including hematopathy, human immunodeficiency virus (HIV), malignant tumors receiving chemotherapy within the previous 3 months, or autoimmune diseases at active stage, (4) diagnosis of fungal infection, (5) diagnosis of viral infection, (6) mild infection and unsatisfaction of the sepsis 3.0 diagnostic criteria.

The study was conducted in accordance with the Declaration of Helsinki (as revised in 2013) and it was approved by the Ethics Committee of Sun Yat-sen Memorial Hospital Affiliated to Sun Yat-sen University (Approval No. SYSKY-2022-281-01). Written consent was obtained from the subject’s authorized representative, due to the subject’s critical illness.

**Figure 1:**
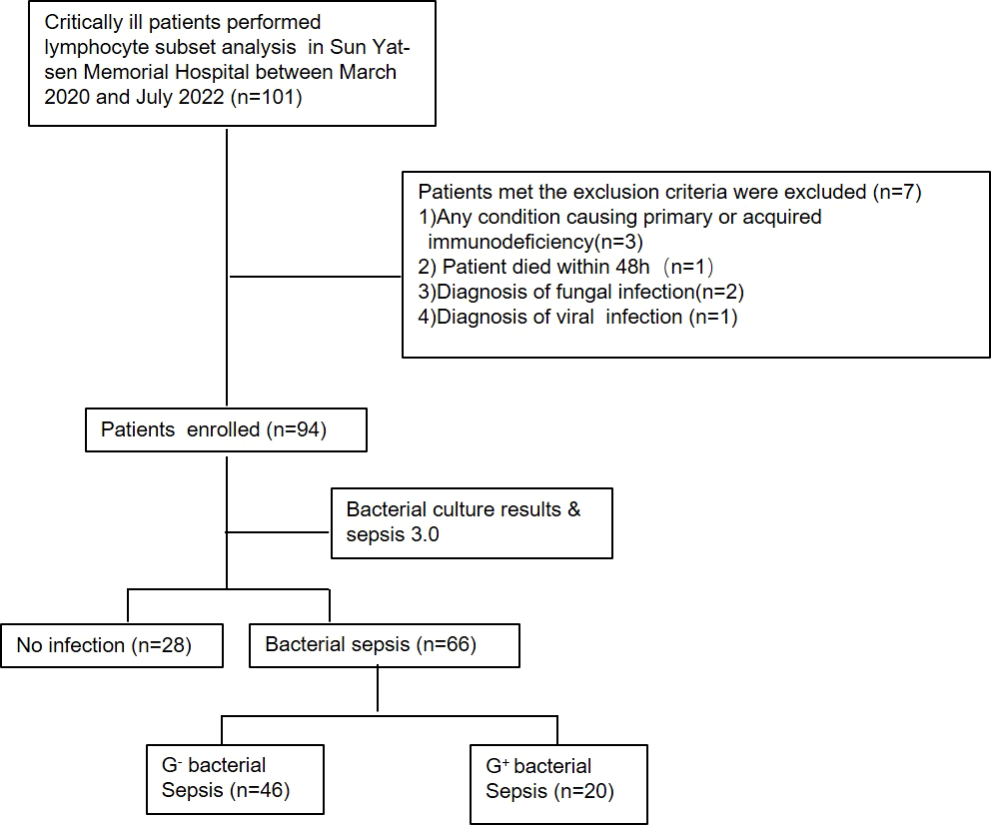
Flowchart depicting patients’ selection.

In the present study, 101 critically ill patients of whom 7 patients were excluded, including 3 patients with hematopathy, 1 patient died within 48 h, 2 patients were diagnosed with fungal infection, and 1 patient was diagnosed with viral infection (**Figure 1**). According to the sepsis 3.0 diagnostic criteria (1), and the results of pathogen culture that isolated from normally sterile sites, including blood, body fluid, and sputum samples aspirated by tracheal intubation, 94 patients were finally included.

### Data collection

Epidemiological data were collected from electronic medical records, including the patients’ age, gender, underlying diseases, and treatments (including supportive treatments and drug therapies). Acute Physiology and Chronic Health Evaluation (APACHE) II and Sequential Organ Failure Assessment (SOFA) scores were calculated on the first day of ICU admission, followed by the analysis of blood lymphocyte subset and cytokines on the first 3 days after ICU admission, and results of the biochemical tests were collected on the same day with lymphocyte subset analysis. The outcomes included the duration of ICU stay, length of hospital stay, and 28-day mortality.

### Multi-color flow cytometry

Muti-color flow cytometry of peripheral blood mononuclear cells (PBMCs) was performed in the Department of Clinical Laboratory, Sun Yat-sen Memorial Hospital, Sun Yat-sen University (Guangzhou, China). Cells were stained for surface markers with the following fluorochrome-conjugated monoclonal antibodies (mAbs): antihuman CD69-PE (FN50), antihuman CD16-PE (B73.1), antihuman CD25-PE-Cy7 (2A3), antihuman CD56-PE-Cy7 (NCAM16.2), antihuman HLA-DR-APC (L243), antihuman CD19-APC-H7 (SJ25C1), antihuman CD8-APC-H7 (SK1), and antihuman CD38-V450 (HB7), which all were purchased from BD Biosciences (Franklin Lakes, NJ, USA); antihuman CD3-FITC (UCHT1), antihuman CD127-PE (R34.34), antihuman CD4-PC5.5 (13B8.2), and antihuman CD45-KO (J33) antibodies were obtained from Beckman Coulter Inc. (Brea, CA, USA). All stained samples were acquired with a 10-color flow cytometer (Navios, Beckman Coulter Inc.), and data were analyzed by Kaluza 2.1.1. software (Beckman Coulter Inc.). The gating strategies are summarized in **Supplementary Figure S1**.

### Cytokine quantification

Cytokines in the patients’ peripheral blood samples were analyzed using a Human Cytometric Bead Array kit (Weimi Bio-Tech Co., Ltd., Beijing, China). Plasma concentrations of 10 cytokines (interleukin-4 (IL-4), IL-5, IL-6, IL-8, IL-10, IL-12, IL-17A, interferon-γ (IFN-γ), tumor necrosis factor-α (TNF-α), and IL-1β) were measured using the Human Cytokine 10-plex Assay kit, according to the manufacturer’s instructions. Briefly, 25 μL of plasma sample was added to each tube. Next, mixed microbeads with dilution of 1:4 (10 μL) were added and the tubes were incubated for 120 min, followed by the incubation with antibodies and Streptavidin-PE, as well as washing after each step. The beads were resuspended in the tube using 150 μL phosphate-buffered saline (PBS), and detected using a FACSCanto II Flow cytometer (BD Biosciences). The data were obtained and analyzed using FCAP Array 3.0 software (BD Biosciences).

### Statistical analysis

The clinical records and laboratory data were collected and analyzed. Comparison was made between no infection group and bacterial sepsis group or G^-^ sepsis subgroup and G^+^ sepsis subgroup, using the Fisher’s exact test or χ^2^ test for categorical variables and the Mann-Whitney U test or Student’s *t*-test for continuous variables. To evaluate the diagnostic sensitivity, specificity, and optimal cutoff value for of each significant continuous variables between G^-^ sepsis and G^+^ sepsis, the receiver operating characteristic (ROC) curve analysis was conducted, and we used the cut-off values to make PCT (>0.53 vs ≤0.53ng/ml) and carbon dioxide combining power (CO^2^CP) (<26.5 vs ≥26.5 mmol/L) as category variables for further logistic regression. Next, we evaluated the diagnostic values of the variables with p<0.05 in the ROC curve and infection sites by performing univariate logistic regression. And the significant variables in univariate logistic regression(p<0.05), as well as the confounders (age and gender) were included to establish the discrimination model for multivariate logistic regression (using method=FSTEP(LR)). Furthermore, the diagnostic performance of the discrimination model was evaluated by ROC curve. For survival analysis, we tested all the variables with a stratified log-rank test of Kaplan–Meier method, and the significant variables with p<0.05 were selected for univariate cox analysis to evaluate their independent prognostic value of outcomes in patients with G^-^ bacterial sepsis. The significant variables in the univariate cox analysis (p < 0.05) were included in a multivariable cox model to estimate the simultaneous effects of prognostic factors on outcomes. The statistical analysis was performed using SPSS 26.0 (IBM Corp., Armonk, NY, USA), Graphpad Prism 8.0 (GraphPad Prism Software Inc., San Diego, CA, USA), and R 4.1.0 (R Development Core Team, R Foundation for Statistical Computing, Vienna, Austria) software. A two-sided P-value less than 0.05 was considered statistically significant.

## Results

### Patients’ characteristics

The enrolled 94 patients were finally divided into no infection group (n=28) and bacterial sepsis group (n=66). Compared with the no infection group, patients in the bacterial sepsis group had higher SOFA and APACHE II scores, and were more likely to receive antibiotics for gram-negative bacteria or gram-positive bacteria, and thymalfasin treatment. Patients in the bacterial sepsis group also had longer ICU stay and hospital stay than those in the no infection group, while the 28-day mortality had no significant difference between the two groups, although there were 30 (45.45%) patients with septic shock and 4 patients died in the bacterial sepsis group (**Table 1**).

**Table 1:** Clinical characteristics of critically ill patients.

	Total(n=94)	No infection (n=28)	Bacterial sepsis (n=66)	p-Value ^a^	Bacterial sepsis (n=66)		
					G^-^ bacterial sepsis (n=46)	G^+^ bacterial sepsis (n=20)	p-Value ^b^
Sex (male/female)	59/35	18/10	41/25	0.843	32/14	9/11	0.059
Age (years)	69(58.75,79.00)	70.5(52.25,79.75)	68 (59,77.25)	0.753	70.50 (59.00,79.00)	65.00 (58.25,73.25)	0.342
sepsis shock (n, %)	30 (31.91%)	0 (0%)	30 (45.45%)	**0.001**	19 (23.91%)	11 (55.00%)	0.304
Comorbidities							
CHD (n, %)	24 (25.53%)	7 (25.00%)	17 (25.76%)	0.939	9 (19.57%)	12 (60.00%)	**0.001**
COPD (n, %)	8 (8.51%)	4 (14.29%)	4 (6.06%)	0.191	4(8.70%)	0 (0.00%)	0.174
Diabetes mellitus (n, %)	14 (14.89%)	2 (7.14%)	12 (18.18%)	0.169	7 (15.22%)	5(25.00%)	0.344
Chronic kidney disease (n, %)	15 (15.96%)	3 (10.71%)	12 (18.18%)	0.366	10 (21.74%)	2 (10.00%)	0.256
Chronic liver disease (n, %)	3 (3.19%)	1 (3.57%)	2 (3.03%)	0.891	2 (4.35%)	0 (0.00%)	0.344
Solid tumor (n, %)	41 (43.62%)	19 (67.86%)	22 (33.33%)	**0.002**	18 (39.13%)	4 (20.00%)	0.13
Stroke (n, %)	15 (15.96%)	4 (14.29%)	11 (16.67%)	0.773	9 (19.57%)	2 (10.00%)	0.338
Charlson comorbidity index	5.00 (4.00,7.00)	5.00 (4.00,6.75)	5.00 (4.00,7.00)	0.967	5.00 (4.00,7.00)	5.00 (4.00,6.75)	0.860
surgical patient (n, %)	66 (70.21%)	24 (85.71%)	42 (63.64%)	**0.032**	29 (63.04%)	13 (65.00%)	0.879
SOFA score	5.00 (3.75,8.00)	4.00 (1.25,4.00)	6 (4,9)	**<0.001**	6.50(4.00,9.25)	5.00 (4.00,6.00)	0.398
APACHE II score	21.00(17.00,25.00)	18.00(12.00,19.75)	22.5 (18,27)	**<0.001**	22.50 (18.00,27.25)	22.5 (18.50,24.00)	0.061
Treatment							
Mechanical ventilation (n, %)	82 (87.23%)	26 (92.86%)	56 (90.91%)	0.287	41 (89.13%)	15 (75.00%)	0.216
RRT (n, %)	20 (21.28%)	3 (10.71%)	17 (25.76%)	0.103	14 (30.43%)	3 (15.00%)	0.188
Other invasive treatment ^$^ (n, %)	15 (15.96%)	2 (7.14%)	13 (19.70%)	0.129	9 (19.57%)	4 (20.00%)	0.967
Noradrenaline(ug/kg/min)	0 (0.00, 0.10)	0 (0,0)	0 (0,0.11)	**0.011**	0 (0,0.13)	0 (0,0.11)	0.748
Glucocorticoid (n, %)	20 (21.27%)	2 (7.14%)	18 (27.27%)	**0.029**	15 (32.61%)	3 (15.00%)	0.14
Gamma globulin (n, %)	33 (35.11%)	4 (14.29%)	29 (43.94%)	**0.006**	21 (45.65%)	8 (40.00%)	0.671
Abs for GPB (n, %)	50 (53.19%)	7 (25.00%)	43(65.15%)	**<0.001**	27 (48.70%)	16 (80.00%)	0.095
Abs for GNB (n, %)	88 (93.62%)	23 (82.14%)	65 (98.48%)	**0.003**	46 (100%)	19 (95.00%)	0.126
Antifungal drugs (n, %)	12 (12.77%)	2 (7.14%)	10 (15.15%)	0.287	9 (19.57%)	1 (5.00%)	0.129
Thymalfasin (n, %)	12 (12.77%)	0 (0.00%)	12 (18.18%)	**0.016**	9 (19.57%)	3 (15.00%)	0.659
Outcomes							
ICU stay (days)	13.50(4.75,21.00)	4.50(2.00,12.50)	17.5 (7.75,26.00)	**<0.001**	17.00 (6.75,27.25)	18.50 (10.50,24.00)	0.889
Hospital stay (days)	27.00(17.00,49.25)	20.00(15.00,25.75)	30.5 (22.75,56)	**<0.001**	30.50 (19.50,60.00)	29.50 (23.00,50.00)	0.861
28-day mortality (death/survivor)	5/89	1/27	4/62	0.623	3/43	1/19	0.812

Data are number of patients (%) or median (interquartile range). CHD, coronary heart disease; COPD, chronic obstructive pulmonary disease; APACHE II, acute physiology and chronic health evaluation II; SOFA, sequential organ failure assessment. RRT, renal replacement therapy; other invasive treatment^$^, including pulse index continuous cardiac output, extra-corporeal membrane oxygenation, intra-aortic balloon pump, high flow or non-invasive ventilation; GNB: gram negative bacteria; GPB: gram positive bacteria.

P-Value ^a^ for the comparison between no infection group and bacterial sepsis group;

p-Value ^b^ for the comparison between G^-^ bacterial sepsis group and G^+^ bacterial sepsis group;

p-Values were estimated by Fisher’s exact test or χ^2^ test and Mann–Whitney U test for categorical variables and continuous variables, respectively.

Bold values was p<0.05.

Among patients in the bacterial sepsis group, there were 46 patients with G^-^ sepsis and 20 patients with G^+^ sepsis. In the G^+^ sepsis subgroup, more patients were complicated with chronic heart disease (CHD), and the other clinical characteristics were similar (**Table 1**). The most common infection site was pulmonary infection (52.17%) in the G^-^ sepsis subgroup, while the infection sites of bloodstream (45.00% vs 17.39%) and wound/soft tissue (25% vs. 6.52%) were more common in the G^+^ sepsis subgroup than those in the G^-^ sepsis subgroup (**Table 2**).

**Table 2:** Infection site of sepsis patients.

Infection site	Bacterial sepsis (n=66)	G^-^ bacterial sepsis (n=46)	G^+^ bacterial sepsis (n=20)	p-Value
Pulmonary infection	27 (40.91%)	24 (52.17%)	3 (15.00%)	**0.005**
Bloodstream infection	17 (25.76%)	8 (17.39%)	9 (45.00%)	**0.018**
Thoracic or abdominal infection	9 (13.64%)	7 (15.22%)	2 (10.00%)	0.57
Wound or soft tissue infection	8 (12.12%)	3 (6.52%)	5 (25.00%)	**0.035**
Other infections	5 (7.58%)	4 (8.70%)	1 (5.00%)	0.602

Data are number of patients (%).

p-Value for the comparison between G^-^ bacterial sepsis group and G^+^ bacterial sepsis group were estimated by Fisher’s exact test.

Bold values was p<0.05.

Patients in the G^-^ sepsis subgroup had higher serum PCT (1.89(0.83,5.01) vs. 0.45(0.22,3.04) ng/ml) and IL-6 (60.19 (25.85,189.63) vs. 32.79 (10.03,100.05) pg/ml) than those in the G^+^ sepsis subgroup, and patients in the G^-^ sepsis subgroup were at a higher risk of metabolic acidosis with a lower serum CO_2_CP level (24.00 (21.00,27.25) vs. 27.50 (24.00,30.75) mmol/L), as well as a lower pH and more negative base excess (BE) in arterial blood gas analysis (**Table 3**), compared with those in the G^+^ sepsis subgroup. While more G^-^ infections happened in the respiratory system, we compared the acid-base variables between pulmonary infection and non-pulmonary infection to investigate the role of infection sites on the acid-base disarranges. As showed in **Supplementary Figure S2A**, we found that among G^-^ septic patients (n=46), CO_2_CP (22.50(19.50,24.50) vs. 26.00(22.25,29.00) mmol/L, p=0.019) and PaCO_2_ (31.10 (27.33, 37.70) vs 38.15 (33.45, 41.23) mmHg, p=0.017) were lower in non-pulmonary infection than pulmonary infection, while both BE (-3.0(-10.5, 5.75) vs. -6.0(-8.7,1.25) mmol/L, p=0.095) and pH (7.44(7.40,7.48) vs. 7.42 (7.40,7.47), p=0.516) were not different between non-pulmonary infection and pulmonary infection. Moreover, PaCO_2_ was also lower (32.90 (30.40, 39.20) vs. 38.50 (34.50, 42.90) mmHg, p= 0.0287) in non-pulmonary infection than pulmonary infection among bacterial sepsis (n=66) (**Supplementary Figure S2B**).

**Table 3:** Inflammatory markers and other markers of critically ill patients.

	Total (n=94)	No infection (n=28)	Bacterial sepsis (n=66)	p-Value ^a^	Bacterial sepsis (n=66)		
					G^-^ bacterial sepsis (n=46)	G^+^ bacterial sepsis (n=20)	p-Value ^b^
Inflammatory markers							
IL-4 (pg/ml)	1.41 (0.81,1.72)	1.54 (0.99,1.77)	1.30 (0.75,1.70)	0.157	1.30 (0.61,1.70)	1.35 (0.95,1.76)	0.499
IL-5 (pg/ml)	1.51 (1.00,2.00)	1.53(1.16,1.99)	1.43 (0.97,2.03)	0.313	1.41 (0.94,2.08)	1.52 (0.98,1.81)	0.922
IL-6 (pg/ml)	58.57(19.41,189.63)	98.37(22.59,318.35)	55.78(14.37,139.54)	0.124	60.19 (25.85,189.63)	32.79 (10.03,100.05)	**0.044**
IL-8 (pg/ml)	17.18 (8.87,29.45)	14.71 (8.65,29.45)	18.54 (9.03,30.11)	0.898	19.42 (9.03,47.41)	14.36(8.17,20.80)	0.14
IL-10 (pg/ml)	4.66 (2.60,8.22)	5.26(2.69,8.54)	4.46 (2.52,8.22)	0.527	5.06 (2.76,9.34)	3.98 (2.14,5.37)	0.068
IL-12 (pg/ml)	1.04 (0.46,1.62)	1.41 (0.37,2.15)	0.90 (0.48,1.48)	0.259	0.92 (0.38,1.48)	0.87 (0.59,1.51)	0.562
IL-17A (pg/ml)	0.9 1 (0.61,1.30)	0.91 (0.79,1.41)	0.79 (0.52,1.28)	0.179	0.89 (0.57,1.76)	0.71 (0.48,0.97)	0.157
IL-1β (pg/ml)	1.02 (0.66,2.01)	1.55 (0.75,2.75)	0.84 (0.60,1.51)	**0.019**	0.84 (0.61,1.98)	0.79 (0.52,1.22)	0.336
TNF-α (pg/ml)	1.05 (0.60,1.76)	1.31 (0.68,2.53)	0.84 (0.57,1.54)	0.059	0.89 (0.54,1.56)	0.82 (0.60,1.45)	0.967
IFN-γ (pg/ml)	1.24 (0.71,1.78)	1.39 (0.76,1.77)	1.24 (0.63,1.78)	0.554	1.26 (0.63,1.62)	1.09 (0.61,1.85)	0.972
hsCRP (mg/L)	60.65 (30,105.35)	77.60(30.60,127.05)	58.30(30.00,100.48)	0.649	56.50 (29.1,105.35)	62.9 (31.58,96.33)	0.949
SAA (mg/L)	178.90(88.70,317.05)	255.72(113.73,320.00)	177.01(59.64,302.85)	0.055	154.22 (43.77,290.27)	220.23 (96.27,315.82)	0.226
PCT (ng/ml)	1.27 (0.34,3.49)	0.87 (0.21,2.65)	1.66(0.40,4.20)	0.153	1.89 (0.83,5.01)	0.45(0.22,3.04)	**0.008**
Arterial blood gas							
pH	7.44±0.05	7.43±0.05	7.45±0.05	**0.0440**	7.43±0.05	7.47± 0.03	**0.002**
PaCO_2_ (mmHg)	35.5 (30.65,39.53)	34.50 (30.35,39.28)	35.75 (30.65,40.20)	0.4040	34.75 (30.48,39.60)	36.60 (32.60,42.85)	0.264
PaO_2_ (mmHg)	122.29±32.55	125.03±37.19	121.13±30.61	0.5990	118.33±32.13	127.58±26.40	0.262
BE (mmol/L)	0 (-4,4)	1 (-3,4.25)	-1.5 (-4,2.5)	0.0720	0 (-5,4)	3.5 (-0.75,8.25)	**0.019**
Lactate (mmol/l)	1.5(1.175,2.30)	1.50 (0.85,2.05)	1.50 (1.20,2.425)	0.3110	1.50 (1.28,2.40)	1.45 (1.13,2.50)	0.812
PaO_2_/FIO_2_	337.10(267.15,398.95)	338.56±114.18	331.17±91.87	0.7410	321.40±98.40	353.63±71.96	0.193
Biochemical markers							
Urea (mmol/L)	9.60(5.80,20.08)	7.00 (4.20,11.68)	12.30 (6.78,26.18)	**0.001**	13.80 (6.55,28.05)	9.30 (6.85,18.70)	0.244
Creatinine (umol/L)	87.50(68.00,128.25)	86.50(71.25,112.25)	92.00(68.00,141.50)	0.71	99.00 (73.00,160.25)	77.50(64.25,124.75)	0.097
CO_2_CP (mmol/L)	24.00(21.00,28.00)	22.00 (20.25,24.75)	24.50 (21.00,29.00)	**0.026**	24.00 (21.00,27.25)	27.50 (24.00,30.75)	**0.026**
Total protein (g/L)	57.54±8.06	56.70± 7.56	57.90± 8.30	0.511	57.60± 7.93	58.58± 9.26	0.665
Albumin (g/L)	32.17±4.84	32.14± 4.37	32.18± 5.06	0.966	32.42± 4.84	31.65± 5.64	0.577
Globulin (g/L)	24.55(22.30,27.5)	24.05 (20.65,25.75)	24.65 (23.45,28.05)	0.19	24.80 (22.18,27.63)	24.45 (23.63,30.75)	0.862
Total bilirubin (μmol/L)	15.40(8.98,27.08)	11.80 (8.38,17.18)	19.40 (10.08,32.15)	**0.009**	22.50 (11.125,33.85)	13.75 (8.88,23.38)	0.169

Data are mean ± standard deviation or median (interquartile range). IL, interleukin; hsCRP, hypersensitive C-reactive protein; SAA, serum amyloid A; PCT, procalcitonin; BE, base excess; CO_2_CP, carbon dioxide combining power.

P-Value ^a^ for the comparison between no infection group and bacterial sepsis group;

p-Value ^b^ for the comparison between G^-^ bacterial sepsis group and G^+^ bacterial sepsis group;

p-Values were estimated by Mann–Whitney U test or t test.

Bold values was p<0.05.

### Bacterial sepsis elevated T cell activation

**Figure 2:**
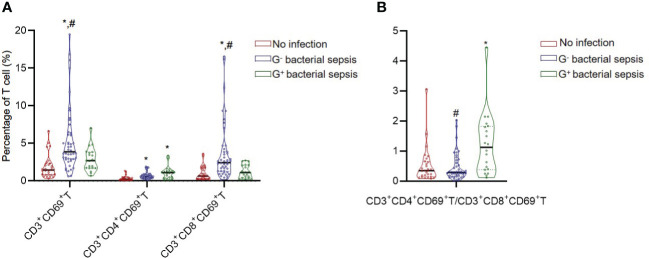
The CD3^+^CD69^+^T cell subset and CD3^+^CD4^+^CD69^+^T/CD3^+^CD8^+^CD69^+^T ratio in critically ill patients. **(A)** The percentages of CD3^+^CD69^+^T subsets in different groups. **(B)** The ratio of CD3^+^CD4^+^CD69^+^T/CD3^+^CD8^+^CD69^+^T in different groups. *, vs. no infection group; #, vs. G^+^ bacterial sepsis group; P<0.05.

The T lymphocyte subset analysis revealed that patients in the bacterial sepsis group had higher percentages of CD3^+^CD38^+^ T cells (13.74 (8.06,24.07) vs. 8.62 (6.23,12.90)) and CD3^+^CD69^+^ T cells ((3.60 (1.96,5.04) vs. 1.46(0.83,2.29)) than those in the no infection group, and further test indicated that percentages of CD3^+^CD8^+^CD38^+^ T, CD3^+^CD8^+^CD69^+^ T, and CD3^+^CD4^+^CD69^+^ T subsets were higher in the bacterial sepsis group than in the no infection group (**Table 4**). Among patients in the bacterial sepsis group, the changes of T lymphocyte subsets between G^-^ sepsis and G^+^ sepsis subgroups were detected, and it was found that patients in the G^-^ sepsis subgroup had higher percentages of CD3^+^CD8^+^T (26.86(16.17, 32.78) vs. 15.62 (11.81, 22.17), P=0.006), CD3^+^CD69^+^ T (3.87(2.90, 6.41)vs. 1.98(1.71, 3.83), P=0.004), CD3^+^CD8^+^HLA-DR^+^ T (23.26(14.26, 34.52) vs. 12.00(8.17, 23.52), P=0.009), and CD3^+^CD8^+^CD69^+^T subsets (2.43(1.30, 3.97)vs. 1.13(0.51, 2.09), P=0.001) compared with those in the G^+^ sepsis subgroup (**Table 4** and **Figure 2A**). However, the ratio of CD3^+^CD4^+^CD69^+^ T/CD3^+^CD8^+^CD69^+^ T was significantly lower in the G^-^ sepsis subgroup than that in the G^+^ sepsis subgroup (0.27(0.15, 0.50) vs. 0.92(0.33,1.73), P<0.001) (**Figure 2B**), although the percentage of CD3^+^CD4^+^CD69^+^ T cells was lower in the G^-^ sepsis subgroup than that in the G^+^ sepsis subgroup, while no significant difference was found (0.60(0.42,0.84) vs. 0.91 (0.42,1.18), P=0.204) (**Table 4**).

**Table 4:** T lymphocyte subpopulation of critically ill patients.

	Total(n=94)	No infection (n=28)	Bacterial sepsis (n=66)	p-Value ^a^	Bacterial sepsis (n=66)		
					G^-^ bacterial sepsis (n=46)	G^+^ bacterial sepsis (n=20)	p-Value ^b^
WBC (cells/ul)	10730(8045,14080)	10185(8842.5,14120)	10975(7727.5,14132.5)	0.801	10730(7070,13662.5)	12860(8292.5,15285)	0.481
Lymphocyte (cells/ul)	795 (457.5,1215)	855 (577.5,1225)	755 (430,1132.5)	0.073	625 (425,1020)	815 (452.5,1510)	0.368
T lymphocyte (cells/ul)	490.766 (288.68, 813.56)	585.16(352.70,891.76)	432.71(256.30,721.11)	0.058	416.40(263.36,659.42)	512.31(231.61,1055.86)	0.615
B lymphocyte (cells/ul)	102.50 (52.91, 204.67)	118.57(74.88,197.52)	95.82(48.89,238.68)	0.346	93.17(40.95,141.19)	165.26(69.80,308.11)	0.06
CD4^+^ T/lymphocyte (%)	39.06(26.99,47.53)	36.88±13.31	37.78±12.50	0.753	36.59±12.97	40.52±11.18	0.243
CD8^+^ T/lymphocyte (%)	21.38(14.59,30.60)	23.44 (15.29, 30.95)	20.93(13.79,30.66)	0.447	26.86 (16.17,32.78)	15.62 (11.81, 22.17)	**0.006**
CD4^+^ T/CD8^+^ T	1.71 (1.09,2.87)	1.47 (1.148, 2.71)	2.00(1.02,3.10)	0.482	1.54 (0.92, 2.62)	2.83 (1.40,4.06)	**0.015**
NK cell (cells/ul)	117.85(60.46,175.13)	140.53(76.77,214.84)	112.01(58.29,166.22)	0.178	116.33(58.67,169.14)	84.08(53.46,156.99)	0.435
Regular NK cell (%)	3.56(1.82,6.05)	3.85(2.02, 5.70)	3.43 (1.55,6.42)	0.546	2.98 (1.55,5.65)	3.98 (1.31, 8.90)	0.606
Killer NK cell (%)	95.69(92.12,97.75)	95.64(93.78,97.26)	95.79(91.16,98.28)	0.632	96.45 (91.98,98.28)	94.28 (90.67, 97.89)	0.236
Treg /lymphocyte (%)	2.69 (2.08,3.39)	2.27 (1.77,3.27)	2.77 (2.16,3.97)	0.197	2.68 (1.76,3.97)	3.10 (2.48, 3.80)	0.147
CD3^+^CD25^+^ T (%)	10.54 (6.38,18.93)	8.02 (5.77,13.43)	11.34(6.71,21.42)	0.054	10.94 (6.33,21.42)	11.39 (9.42, 22.13)	0.443
CD3^+^CD69^+^ T (%)	2.89 (1.50,4.77)	1.46 (0.83,2.29)	3.60 (1.96,5.04)	**<0.001**	3.87 (2.90,6.41)	1.98 (1.71,3.83)	**0.004**
CD3^+^HLA-DR^+^ T (%)	36.97(27.20,53.65)	36.88(26.45,55.90)	37.21(27.53,53.65)	0.878	38.93 (29.78,54.34)	29.21 (23.10,48.02)	0.063
CD3^+^CD38^+^ T (%)	11.44(7.17,19.43)	8.62 (6.23,12.90)	13.74 (8.06,24.07)	**0.027**	15.84 (8.41,27.14)	9.65 (6.48,16.91)	0.081
CD3^+^CD8^+^HLA-DR^+^ T (%)	19.94(11.78,32.24)	19.77(12.35,27.71)	20.02(11.59,32.54)	0.914	23.26 (14.26,34.52)	12.00 (8.17,23.52)	**0.009**
CD3^+^CD4^+^CD25^+^ T (%)	8.32 (5.73,15.72)	7.22 (5.26,10.73)	9.85 (6.28,18.34)	0.050	8.72 (5.73,18.56)	10.27 (7.88,18.53)	0.477
CD3^+^CD4^+^CD38^+^ T (%)	5.42 (3.375,8.145)	5.34 (3.025,7.89)	5.46 (3.54,8.34)	0.467	5.57 (3.54,8.34)	5.46 (3.32,9.47)	0.878
CD3^+^CD4^+^CD69^+^ T (%)	0.49 (0.28,0.90)	0.23 (0.13,0.47)	0.61 (0.42,1.01)	**<0.001**	0.60 (0.42,0.84)	0.91 (0.42,1.18)	0.204
CD3^+^CD4^+^HLA-DR^+^ T (%)	12.88(9.22,15.66)	11.21(9.19,16.08)	12.95 (9.21,15.58)	0.704	12.12 (9.05,15.58)	13.78(11.22,15.75)	0.364
CD3^+^CD8^+^CD25^+^ T (%)	0.38 (0.13,0.94)	0.35 (0.12,0.86)	0.38 (0.13,0.99)	0.652	0.35 (0.09,1.08)	0.71 (0.30,0.96)	0.123
CD3^+^CD8^+^CD38^+^ T (%)	3.78 (1.84,11.32)	2.89 (1.15,4.71)	4.47 (1.91,13.73)	**0.035**	8.66 (1.89,16.85)	2.87 (1.99,4.88)	0.073
CD3^+^CD8^+^CD69^+^ T (%)	1.55 (0.63,2.64)	0.66 (0.30,1.72)	2.00 (0.94,3.02)	**<0.001**	2.43 (1.30,3.97)	1.13 (0.51,2.09)	**0.001**
CD3^+^CD4^+^CD69^+^ T/ CD3^+^CD8^+^CD69^+^ T	0.32 (0.19,0.76)	0.35 (0.16,0.65)	0.32 (0.19,0.87)	0.830	0.27 (0.15, 0.50)	0.92 (0.33,1.73)	**<0.001**

Data are median (interquartile range) or mean± standard deviation. WBC: white blood cell; NK cell: nature killer cell.

P-Value ^a^ for the comparison between no infection group and bacterial sepsis group;

p-Value ^b^ for the comparison between G^-^ bacterial sepsis group and G^+^ bacterial sepsis group;

p-Values were estimated by Mann–Whitney U test or t test.

Bold values was p<0.05.

As pneumonia is the primary source of sepsis and our data indicated more G^-^ infections (24/46, 52.7%) happened in the respiratory system, we further investigated whether pulmonary infection affect peripheral lymphocyte subset in sepsis patients. The results indicated that all the lymphocyte subset ratios were not different between pulmonary infection and non-pulmonary infection among G^-^ septic patients (**Supplementary Table S1**). However, among the bacterial septic patients (including G^-^ and G^+^ sepsis), CD3^+^CD8^+^HLA-DR^+^ T (%) was significantly higher in pulmonary infection than non-pulmonary infection (25.32 (16.12, 33.09) vs. 14.28 (8.21, 32.36) %, p=0.036) (**Supplementary Table S2**).

### Diagnostic biomarkers for G^-^ sepsis (versus G^+^ sepsis)

**Figure 3:**
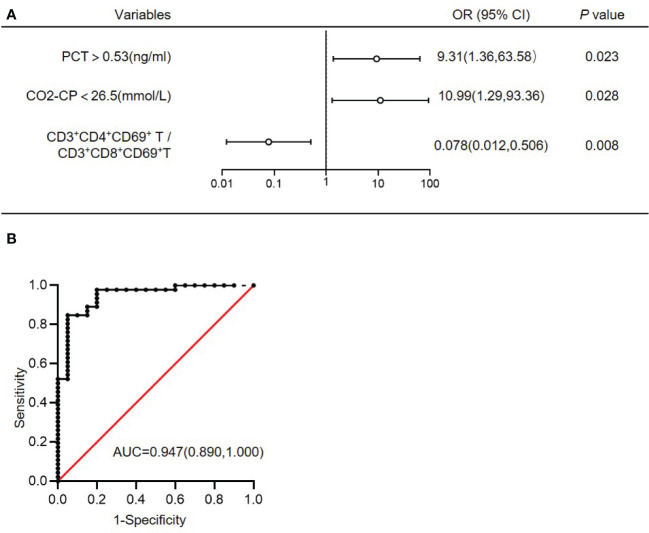
Multivariate logistic regression analysis of G^-^ bacterial sepsis (versus G^+^ bacterial sepsis). **(A)** Multivariate logistic regression analysis of G^-^ bacterial sepsis (versus G^+^ bacterial sepsis). **(B)** ROC curve of the multivariate logistic regression model. AUC, area under the ROC curve; OR, odds ratio; 95%CI, 95% confidence interval; CO_2_CP, carbon dioxide combining power; PCT, procalcitonin.

ROC curve analysis was performed to estimate the value of each significant continuous variables between G^-^ sepsis and G^+^ sepsis in the diagnosis of G^-^ sepsis (versus G^+^ sepsis), and the results displayed that the percentages of CD3^+^CD8^+^T, CD3^+^CD69^+^T, CD3^+^CD8^+^HLA-DR^+^T, and CD3^+^CD8^+^CD69^+^T, the ratios of CD4^+^T/CD8^+^T and CD3^+^CD4^+^CD69^+^T/CD3^+^CD8^+^CD69^+^ T, PCT, and CO_2_CP could welly predict the occurrence of G^-^ sepsis (versus G^+^ sepsis), as shown in **Table 5**, and we used the cut-off values to make PCT (>0.53 vs ≤0.53ng/ml) and CO_2_CP(<26.5 vs ≥26.5 mmol/L= as category variables for further logistic regression. The results of multivariate logistic regression analysis (including the significant variables in univariate logistic analysis (p<0.05, **Supplementary Table S3**), as well as the confounders (age and gender)) showed that infection sites (P=0.061), PCT>0.53ng/ml (odds ratio (OR)=9.31(1.36,63.58); P=0.023), CO2CP<26.5 mmol/l (OR=10.99(1.29,93.36); P=0.028), and CD3^+^CD4^+^CD69^+^T/CD3^+^CD8^+^CD69^+^T ratio (OR=0.078(0.012,0.506); P=0.008) were associated with G^-^ sepsis (**Figure 3A** and **Supplementary Table S3**), and the area under the ROC curve (AUC) of the multivariable logistic regression model in predicting G^-^ sepsis (versus G^+^ sepsis) was 0.947 (**Figure 3B**).

**Table 5:** The AUROC and cut-off points in prediction of G^-^ bacterial sepsis (vs G^+^ bacterial sepsis).

Variables	Cut-off point	AUROC (95%CI)	Sensitivity	Specificity	p-Value
CD3^+^CD8^+^T (%)	26.66	0.71 (0.58, 0.84)	0.522	0.900	**0.006**
CD4^+^ T/CD8^+^ T	2.00	0.69 (0.55, 0.83)	0.609	0.750	**0.015**
CD3^+^CD69^+^ T (%)	1.99	0.72 (0.60, 0.85)	0.848	0.550	**0.004**
CD3^+^CD8^+^HLA-DR^+^ T (%)	14.33	0.70 (0.56, 0.84)	0.761	0.650	**0.009**
CD3^+^CD8^+^CD69^+^ T (%)	2.19	0.77 (0.65, 0.88)	0.587	0.850	**0.001**
CD3^+^CD4^+^CD69^+^ T/ CD3^+^CD8^+^CD69^+^ T	0.36	0.77 (0.65,0.90)	0.696	0.750	**<0.001**
APACHE II score	24.50	0.57 (0.42,0.71)	0.413	0.85	0.399
IL-6 (pg/ml)	27.96	0.63 (0.59,0.77)	0.761	0.500	0.098
IL-10 (pg/ml)	5.84	0.63 (0.49,0.77)	0.435	0.750	0.104
PCT (ng/ml)	0.53	0.70 (0.57,0.84)	0.804	0.600	**0.009**
Creatinine (umol/L)	92.00	0.63 (0.49,0.77)	0.587	0.700	0.097
CO_2_CP (mmol/L)	26.50	0.67 (0.54,0.81)	0.739	0.550	**0.027**

AUROC, area under the ROC curve; 95%CI, 95% confidence interval;

APACHE II, acute physiology and chronic health evaluation II; IL, interleukin;

PCT, procalcitonin; CO_2_CP, carbon dioxide combining power.

Bold values was p<0.05.

### The prognostic role of T cell profiles for patients with G^-^ sepsis

As SOFA score, platelet, and acidosis are efficacious marker for predicting the prognosis of septic patients, we investigated their correlations with lymphocyte subsets. We found that SOFA score was positive correlated with CD3^+^CD69^+^ T (%), CD3^+^CD4^+^CD69^+^ T (%), and CD3^+^CD8^+^CD69^+^ T (%), both in bacterial sepsis group(n=66) (**Supplementary Figure S3A**) and G^-^ bacterial sepsis(n=46) (**Supplementary Figure S3B**), however, in G^+^ bacterial sepsis, SOFA score was positive correlated with Treg (%) and negative correlated with CD3^+^CD8^+^T (%) (**Supplementary Figure S3C**). Although the platelet count was not significantly different between sepsis group and no infection group, or between G^-^ sepsis and G^+^ sepsis (**Supplementary Figure S4A**), however, the platelet count was negatively correlated with CD3^+^CD69^+^T(%) and CD3^+^CD8^+^CD69^+^T(%) and positively correlated with CD3^+^CD4^+^CD69^+^T/CD3^+^CD8^+^CD69^+^ T, in bacterial sepsis group (n=66) (**Supplementary Figure S4B**) and G^-^ sepsis (n=46)(**Supplementary Figure S4C**), but not in G^+^ sepsis group (n=20) (**Supplementary Figure S4D**). The analysis of correlations of acid-base abnormalities with lymphocyte subset showed that both CO_2_CP and BE were negatively correlated with WBC in bacterial sepsis, while pH was negatively correlated with CD3^+^CD8^+^T (%) (r=-0.2879, p=0.0191) (**Supplementary Figure S2C**). These data indicated that SOFA and platelet were correlated with CD69 activation in sepsis, especially in G^-^ sepsis.

**Figure 4:**
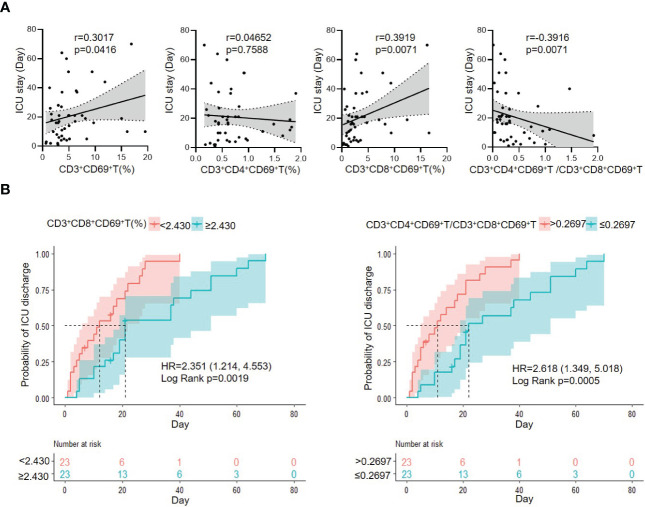
The correlation of CD3^+^CD69^+^T cell subset with ICU stay, and Kaplan–Meier analysis of probability of ICU discharge according to CD3^+^CD69^+^T cell subsets in G^-^ bacterial sepsis group. **(A)** Correlation of ICU stay and CD3^+^CD69^+^T cell subsets in G^-^ bacterial sepsis group. **(B)** Kaplan–Meier analysis of probability of ICU discharge according to CD3^+^CD69^+^T cell subset in G^-^ bacterial sepsis group. HR, hazard ratio; 95%CI, 95% confidence interval.

As the CD3^+^CD69^+^T subsets were changed obviously in G^-^ sepsis, we further investigate the prognostic role of CD3^+^CD69^+^T subsets in ICU discharge for patients with G^-^ bacterial sepsis. The correlation of ICU stay with CD3^+^CD69^+^T subsets was firstly analyzed, and it was found that ICU stay was positively correlated with CD3^+^CD69^+^T (r=0.3017, P=0.0416) and CD3^+^CD8^+^CD69^+^T subsets (r=0.3919, P=0.0071), and negatively associated with the ratio of CD3^+^CD4^+^CD69^+^ T/CD3^+^CD8^+^CD69^+^ T (r=-0.3916, P=0.0071) in patients with G^-^ bacterial sepsis, while did not significantly correlate with CD3^+^CD4^+^CD69^+^T subsets (**Figure 4A**). In patients with G^-^ bacterial sepsis, the Kaplan-Meier analysis revealed that CD3^+^CD8^+^CD69^+^T subsets accounted for ≥2.43% (median: 21 vs. 12 days; hazard ratio (HR)= 2.351 (1.214,4.553); P=0.0019) and the ratio of CD3^+^CD4^+^CD69^+^ T/CD3^+^CD8^+^CD69^+^ T accounted for ≤0.2697 (median: 22 vs. 11 days; HR=2.618 (1.349,5.018); P=0.0005), were both significantly associated with a poor ICU discharge (**Figure 4B**). The univariate Cox regression analysis revealed that G^-^ bacterial septic patients with CD3^+^CD4^+^CD69^+^T/CD3^+^CD8^+^CD69^+^ T ratio ≤0.2697, CD3^+^CD8^+^HLA-DR^+^T>23.255%, and Charlson comorbidity index (CCI)>5 had a poor ICU discharge, and the multivariable Cox regression analysis showed that CD3^+^CD4^+^CD69^+^T/CD3^+^CD8^+^ CD69^+^T ratio ≤0.2697 was an independent prognostic factor for the ICU discharge in G^-^ bacterial septic patients (HR: 0.34 (0.13, 0.88), P=0.026) (**Table 6**).

**Table 6:** Cox regression analysis of factors associated with the probability of ICU discharge for G^-^ sepsis patients.

Variables	Category	Univariabale cox regression			Multivariable cox regression		
		HR	95%CI	P.value	HR	95%CI	P.value
CD3^+^CD4^+^CD69^+^T/CD3^+^CD8^+^CD69^+^T	≤0.2697 vs >0.2697	0.31	0.16-0.62	**0.001**	0.34	0.13-0.88	**0.026**
CD3^+^CD8^+^HLA-DR^+^T (%)	>23.255 vs ≤23.255	0.48	0.24-0.96	**0.036**	1.06	0.44-2.59	0.895
Charlson comorbidity index	>5.0 vs ≤5.00	0.50	0.25-1.00	**0.049**	0.70	0.34-1.46	0.345
CO_2_CP (mmol/L)	<24.00 vs ≥24.00	1.31	0.69-2.46	0.411			
PCT (ng/ml)	>1.79 vs ≤1.79	1.03	0.55-1.93	0.928			
Sepsis shock	yes vs no	1.02	0.53-1.95	0.952			
SOFA	>6.5 vs≤6.5	0.8	0.43-1.48	0.477			
Thamalfasin treatment	yes vs no	0.46	0.2-1.06	0.069			
Age	10-year increments	0.86	0.72-1.04	0.118			

HR, hazard ratio; 95%CI, 95% confidence interval; CO_2_CP, carbon dioxide combining power; PCT, procalcitonin;

SOFA, sequential organ failure assessment.

Bold values was p<0.05.

There was no significant prognostic role of T cell profile in 28-day mortality, which may be due to the few non-survival cases (n=3 in 46 G^-^ sepsis). The cox analysis based on hospital day for G^-^ sepsis patients showed that CD3^+^CD8^+^CD69^+^T (%) and CD3^+^CD8^+^CD38^+^T (%) were associated with the hospital discharge probability in univariable cox regression, however, there was not significant predictive value of the hospital discharge probability in multivariable cox regression (**Supplementary Table S4**).

## Discussion

Sepsis is a life-threatening complication, and bacteria are the most common cause of sepsis (11). A rapid discrimination of G^-^/G^+^ pathogens enable appropriate antibiotic treatment, and could in turn improve patients’ survival. In the present study, we illustrated a novel discrimination model of the low ratio of CD3^+^CD4^+^CD69^+^T/CD3^+^ CD8^+^CD69^+^T, PCT>0.53 ng/ml, and CO_2_CP<26.5 mmol/L, which could strongly predict the diagnosis of the G^-^ sepsis (versus G^+^ sepsis), with an AUC of 0.947. Importantly, it was found that CD3^+^CD4^+^CD69^+^T/CD3^+^CD8^+^CD69^+^T ratio ≤0.2697, CD3^+^CD8^+^HLA-DR^+^T>23.255%, and CCI >5 were significantly associated with the poor ICU discharge, and CD3^+^CD4^+^CD69^+^T/CD3^+^CD8^+^CD69^+^T ratio was an independent prognostic factor for the ICU discharge in G^-^ septic patients.

Lymphocytes play a key role in initiating and propagating the septic response as they orchestrate the interaction between the innate and adaptive immune responses (23). However, there have been limited researches on the role of functional subsets of lymphocytes in the diagnosis of G^-^ sepsis (versus G^+^ sepsis) in critically ill patients. Herein, we monitored the activation markers of CD25, CD38, CD69, and HLA-DR in T cell subsets in early septic patients. Our data indicated that the level of HLA-DR in CD3^+^CD8^+^T subset was remarkably elevated in G^-^ sepsis (versus G^+^ sepsis), whereas there was no significant difference in expression levels of CD25 and CD38 in CD3^+^/CD3^+^CD4^+^/CD3^+^CD8^+^T subset, as well as HLA-DR levels in CD3^+^/CD3^+^CD4^+^T subsets, between patients with G^-^/G^+^ bacterial sepsis. There are controversies on the changes of CD3^+^HLA-DR^+^ expression during sepsis (24, 25). The different expression levels of HLA-DR in T cells could contribute to the different sites or pathogens of underlying infection and disease severity, as indicated by Gogos et al. (26) who reported that the HLA-DR expression was significantly reduced in severe septic patients with intraabdominal and pyelonephritis infections. And interestingly enough, our data displayed more G^-^ infections happened in the respiratory system, and we found that CD3^+^CD8^+^HLA-DR^+^ T (%) was significantly higher in pulmonary infection than non-pulmonary infection among the septic patients. We therefore speculated that the enhanced activation of CD3^+^CD8^+^HLA-DR^+^T cell may particularly regulate the inflammatory response to pulmonary infection in septic patients. This was in line with previous studies (27, 28) documented that the specific infection site of sepsis triggers distinct differences in host immune response.

Importantly, we found that the CD69 level in T subsets was altered observably according to the subpopulation of underlying bacterial sepsis. Our finding revealed that the percentage of CD3^+^CD69^+^ T and CD3^+^CD8^+^CD69^+^ T subset were higher in the bacterial sepsis group (versus the no infection group), and an even higher in the G^-^ sepsis subgroup than that in the G^+^ sepsis subgroup. Additionally, septic patients with G^-^ sepsis had a noticeably lower ratio of CD3^+^CD4^+^CD69^+^T/CD3^+^CD8^+^CD69^+^T than that in the G^+^ sepsis subgroup. Our results are consistent with Liao et al.’s findings (29), which suggested that the amount of CD69^+^ γδ T cells was larger in septic patients than that in healthy controls; and septic patients with poor outcomes had impaired CD69 expression level in γδ T cells after stimulation of pamidronate (29), indicating that CD69, as an early activation biomarker of T cells, could play a crucial role in the early of sepsis. CD69 marker has exhibited an active immunophenotype of circulating immune effector cells not only in bacterial sepsis, but also in fungal sepsis (i.e., patients with candidemia have increased CD69 expression levels in CD8^+^T cells compared with healthy controls, while without different CD69 expression levels in CD4^+^T cells) (30). Interestingly, both high SOFA score and low platelet count were found to be positively correlated with CD3^+^CD69^+^T (%) and CD3^+^CD8^+^CD69^+^ T (%) in G^-^ bacterial sepsis, but not in G^+^ sepsis, while there was no significant correlation with CD3^+^CD4^+^CD69^+^ T (%), which indicating that CD3^+^ CD8^+^CD69^+^ T may be a reliable biomarker of G^-^ sepsis.

The multivariate logistic regression model not only included the T activation profiles, but also integrated with clinical evaluations (CO_2_CP, PCT, and infection sites) in differentiating the subpopulations of bacterial sepsis. Given that the pH, CO_2_CP, and BE were relatively lower in the G^-^ septic patients than G^+^ septic patients, and 52.17% G^-^ sepsis happened in the respiratory system, we further investigated the role of infection site on the acid-base disarranges. We found that in G^-^ sepsis, CO_2_CP and PaCO_2_ were lower in non-pulmonary infection than pulmonary infection, but there were no statistical differences in pH and BE between the two subgroups. These results demonstrated that low CO_2_CP, 95% of which form from serum bicarbonate (31), may be more purely revealing the metabolic acidosis than pH in the non-pulmonary infection subgroup that excluding the impact of pneumonia, while pH is affected by both of the respiratory and metabolic abnormalities (32). Moreover, we speculate the respiratory alkalosis in non-pulmonary infection subgroup is a compensatory mechanism for metabolic acidosis (32),while respiratory acidosis in pulmonary infection subgroup is caused by hypercapnic respiratory failure and the required lung-protective strategy using low tidal volumes used for them (33, 34). As immunological activation is intimately linked to acidosis occurrence (35), and acid-base changes are also proved to influence a wide range of immune functions (36, 37). We observed both CO_2_CP and BE were negatively correlated with WBC, and pH was negatively correlated with CD3^+^CD8^+^T (%). However, acid-base variables did not correlate with CD3^+^CD69^+^T subsets in sepsis, indicating acid-base changes didn’t affect CD3^+^CD69^+^T subsets ratios. Acidotic pH microenvironments are proved to damage tumor-specific CD8^+^T lymphocytes function (38), However, the minor pH variation with significant difference (7.43 ± 0.05 vs. 7.47± 0.03) between G^-^/G^+^ may not be sufficient to affect peripheral T subset ratios. Therefore, we supposed that it was sepsis, but not sepsis-induced acid-base alterations, triggered the peripheral CD3^+^CD69^+^T subsets changes.

The prognosis of sepsis is mainly influenced by patients’ characteristics (e.g., age, immunological status, and comorbidities) (39–41) and infectious characteristics (e.g., pathogen type, virulence, and site of infection) (39, 42, 43). In the present study, the changes in T activation profiles have been found to be correlated with the outcomes of septic patients. We found that CD3^+^CD4^+^CD69^+^T/CD3^+^CD8^+^CD69^+^T ratio ≤ 0.2697, CD3^+^CD8^+^HLA-DR^+^T >23.255%, and CCI >5 were significantly associated with the poor ICU discharge, and CD3^+^CD4^+^CD69^+^T/CD3^+^CD8^+^CD69^+^T ratio was noted as an independent prognostic factor for ICU discharge. The prognostic role of low level CD3^+^CD4^+^CD69^+^T/CD3^+^CD8^+^CD69^+^T ratio in G^-^ sepsis may be similar to that of low level CD4^+^T/CD8^+^T ratio in sepsis in previous studies, which may reflect the imbalance of T suppression/activation of adaptive immune response during pathophysiologic process in sepsis (44, 45). In G^-^ sepsis (versus G^+^ sepsis), we observed that both CD3^+^CD69^+^T and CD3^+^CD8^+^CD69^+^T (%) were remarkably upregulated, while CD4^+^T/CD8^+^T ratio, CD3^+^CD4^+^CD69^+^T (%) and CD3^+^CD4^+^T were downregulated (although the latter two indexes without significant difference). The low level of CD3^+^CD4^+^CD69^+^T/CD3^+^CD8^+^CD69^+^T ratio in G^-^ sepsis (versus G^+^ sepsis) was mainly due to the upregulated CD3^+^CD8^+^CD69^+^ T (%) (CD8^+^T cell activation) and in part a consequence of the relatively loss of CD4^+^T(%)(CD4^+^T suppression). Thus, the low level CD3^+^CD4^+^CD69^+^T/CD3^+^CD8^+^CD69^+^T ratio in G^-^ sepsis may display an adaptive immune state of excessive CD8^+^T activation and early occurrence of CD4^+^T suppression resulting from G^-^ sepsis and correlate with the poor prognosis of G^-^ sepsis.

In the multivariate logistic regression model, T cell profiles facilitate the faster discrimination of the underlying pathogens in sepsis than the traditional pathogen culture, and show the following features and benefits: (i) Peripheral blood samples could be easily collected; (ii) Rapid detection (within 3 hours) benefits early diagnosis (AUC 0.947); (iii) Offer a great value in the prognosis of G^-^ sepsis. Furthermore, CD3^+^CD8^+^CD69^+^T could be a target of immunomodulation intervention in G- sepsis, and the adjustment of immunological status could be one of the most valuable approaches to improve the prognosis. There are also several limitations in our study. Firstly, the sample size was limited in this study and the findings needed to be further validated in a large-scale study. Secondly, the study with a confined infection source should be further conducted to investigate the value of infection site on differentiating G^-^/G^+^ sepsis and its relationship with the T cell profiles. Thirdly, we used a single time point to conduct our prospective, observational study. The dynamic changes of pro-inflammatory and immunosuppression markers at multiple time points in sepsis need to be further explored.

## Conclusions

The present study aimed to detect the changes of T lymphocyte subsets responding to different pathogens of bacterial sepsis, as well as exploration of their diagnostic and prognostic values. In summary, it was found that compared with patients with G^+^ sepsis, patients with G^-^ sepsis exhibited a higher percentage of CD3^+^CD8^+^CD69^+^T cells and a noticeably lower CD3^+^CD4^+^CD69^+^T/CD3^+^CD8^+^CD69^+^T ratio. Moreover, the integrated profile of the lower CD3^+^CD4^+^CD69^+^T/CD3^+^CD8^+^CD69^+^T ratio, PCT>0.53 ng/ml, and CO_2_CP<26.5 mmol/l was favored to the differentiation of G^-^ sepsis from G^+^ sepsis, with an AUC of 0.947 in the regression model. Importantly, the lower CD3^+^CD4^+^CD69^+^T/CD3^+^CD8^+^CD69^+^T ratio (≤0.2697) was independently associated with the poor ICU discharge in patients with G^-^ bacterial sepsis, indicating that the elevated activation of CD3^+^CD8^+^CD69^+^T cells may regulate the excessive inflammatory response of G^-^ sepsis, thereby resulting in the poor prognosis.

## Data availability statement

The original contributions presented in the study are included in the article/**Supplementary Material**. Further inquiries can be directed to the corresponding authors.

## Ethics statement

The studies involving human participants were reviewed and approved by the Ethics Committee of Sun Yat-sen Memorial Hospital Affiliated to Sun Yat-sen University (Approval No. SYSKY-2022-281-01). The patients/participants provided their written informed consent to participate in this study.

## Author contributions

YX and CH contributed to the study conception and design. YX, LP and YZ contributed to the development of methodology. CH, HX, WeiL, and WenL contributed to acquisition of data. CH, YX, HX and MZ performed the data analysis and wrote the manuscript. YX and MZ participated in revising the manuscript. All authors contributed to the article and approved the submitted version.
