# Supplementary material for: T cell activation profiles can distinguish gram negative/positive bacterial sepsis and are associated with ICU discharge
Source: Front Immunol. 2023 Jan 10;13:1058606. doi: 10.3389/fimmu.2022.1058606 (PMC9871918; doi:10.3389/fimmu.2022.1058606)
Supplement: Supplementary file 1 [file DataSheet_1.pdf]

Supplementary Materials for

T cell activation profiles can distinguish gram negative/positive bacterial sepsis and are associated with ICU discharge

Canxia Huang[#], Hui Xiong[#], Weichao Li, Lu Peng, Yukai Zheng, Wenhua Liao, Minggen Zhou[*], Ying Xu[*]

*Corresponding authors: xuying49@mail.sysu.edu.cn; zhoumg@mail.sysu.edu.cn

This PDF file includes:

Supplementary Figure S1 to S4
Supplementary Table S1 to S4

Figure S1

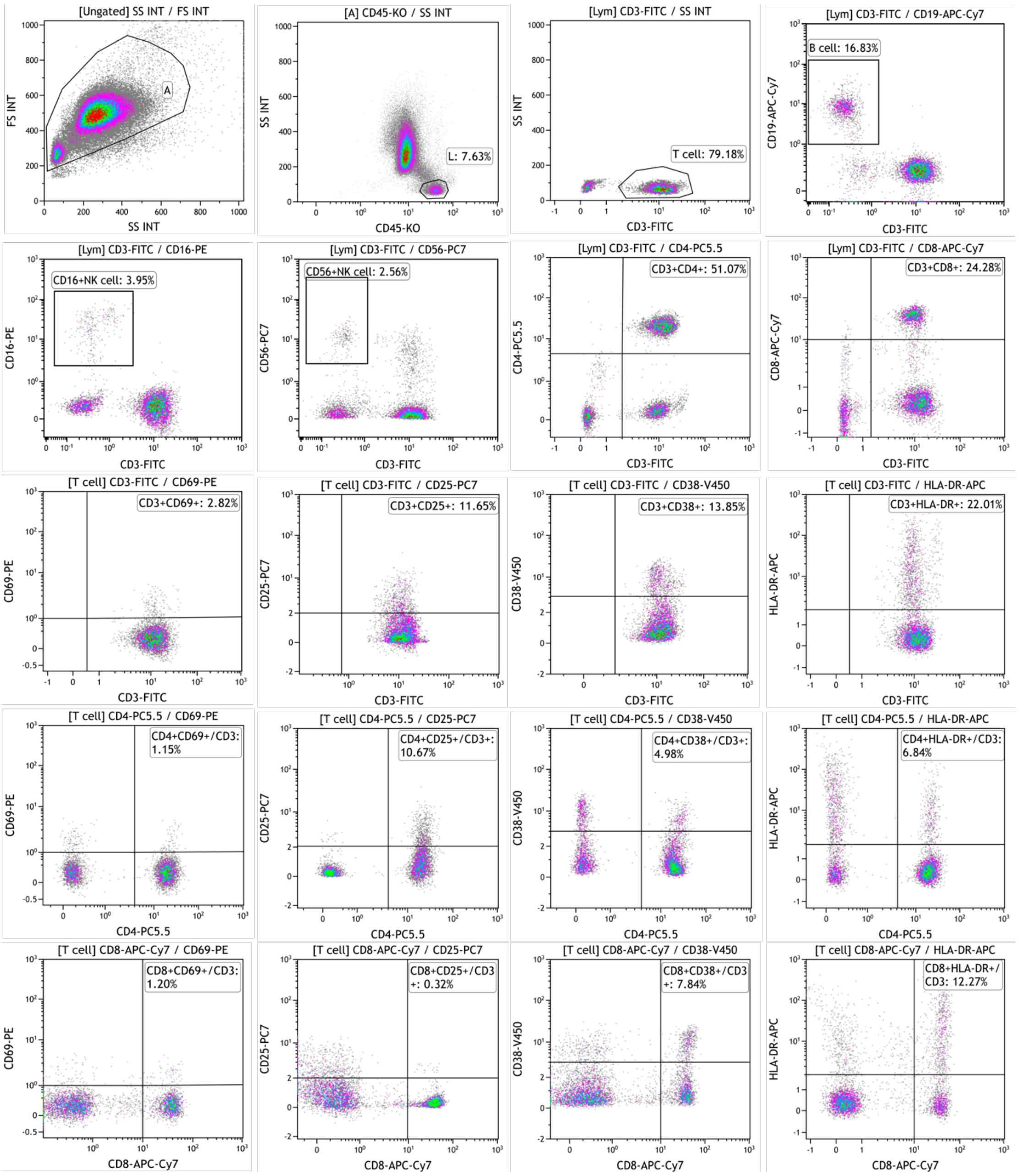

Supplementary Figure S1. Representative flow histograms of lymphocyte gating strategy.

PBMCs from a septic patient were immunostained for T, B, NK, CD4+T, CD8+T cells and T activation profiles. The lymphocyte fraction in the PBMCs was identified by characteristic side scatter properties and CD45, and T, B, NK, CD4+T, CD8+T cells identified by cell-specific antibodies of CD3, CD19, CD16, CD56, CD4, CD8. Activation markers of CD69, CD25, CD38, HLA-DR were stained on T cell, CD4+T and CD8+T subsets, respectively.

Figure S2

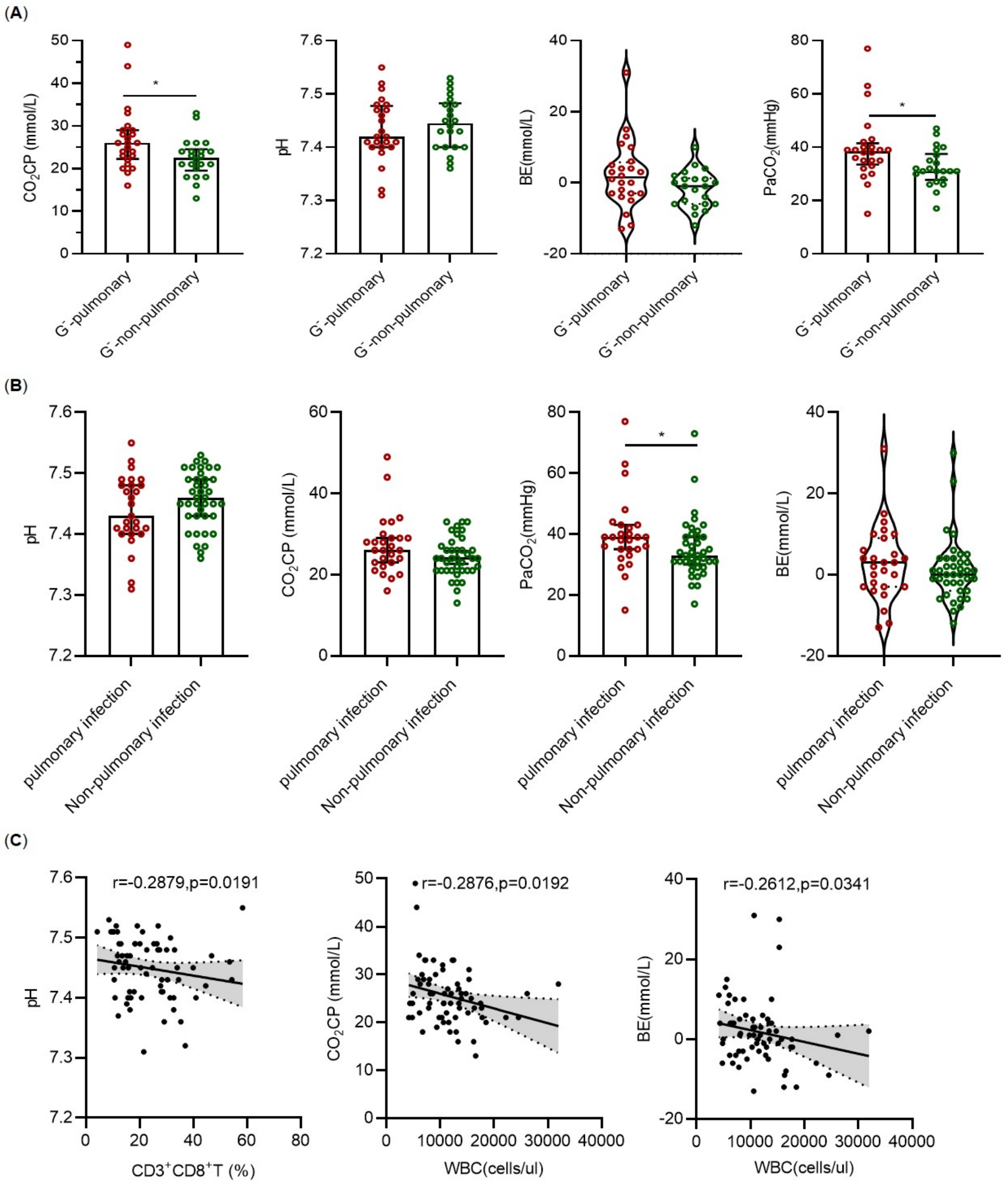

Supplementary Figure S2. The relationship of acid-base variables with pulmonary infection and the lymphocyte subset ratios. (A) The comparisons of acid-base variables between pulmonary infection and non-pulmonary infection in G^- septic patients (n=46). (B) The comparison of acid-base variables between pulmonary infection and non-pulmonary infection in bacterial septic patients(n=66). (C) The correlations of acid-base variables with lymphocyte subset in bacterial septic patients (n=66). *, p<0.05.

Figure S3

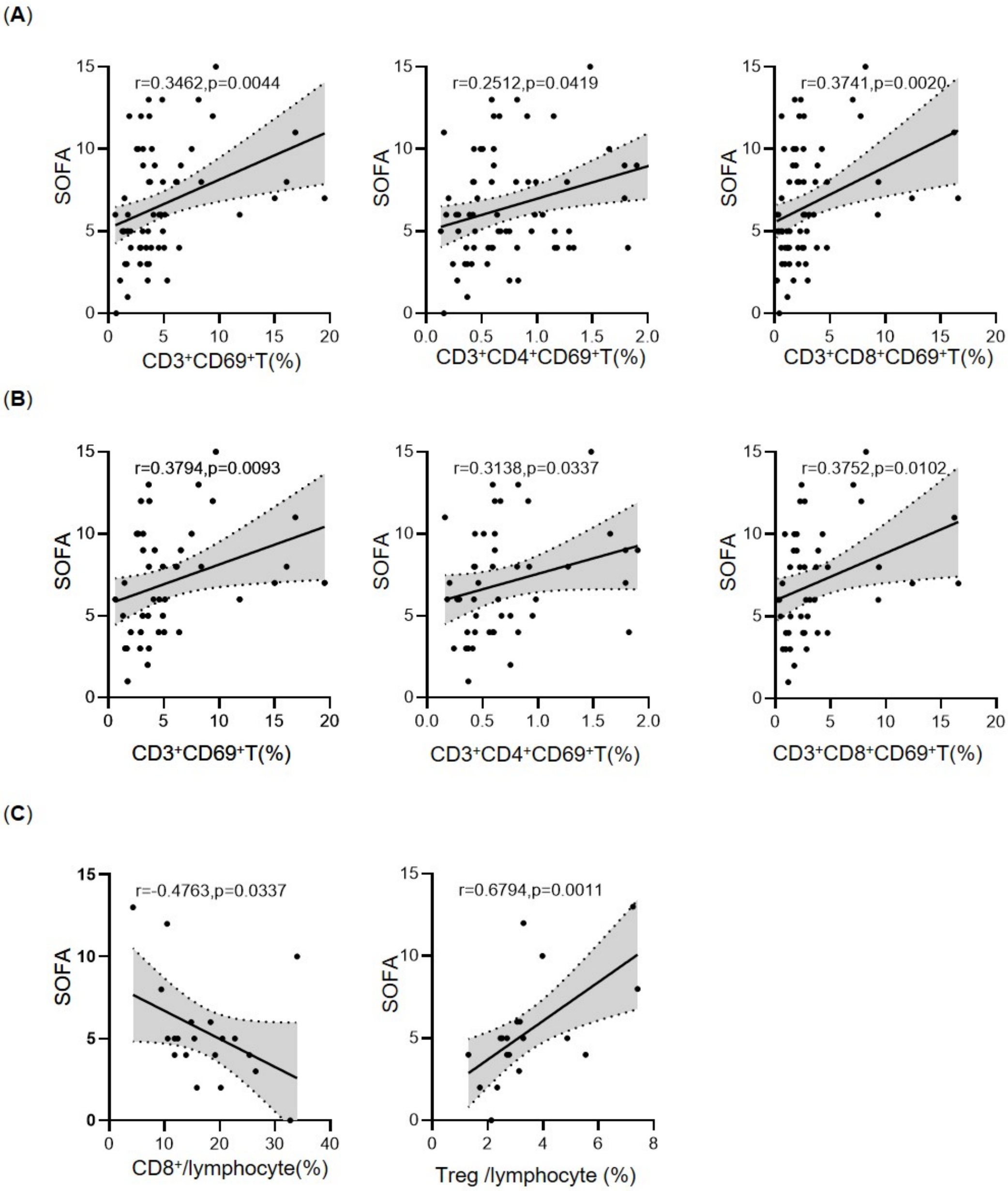

Supplementary Figure S3. The correlation of SOFA score with lymphocyte subset.

(A) Correlation of SOFA score with lymphocyte subset in bacterial sepsis group (n=66). (B) Correlation of SOFA score with lymphocyte subset in G^- sepsis group (n=46). (C) Correlation of SOFA score with lymphocyte subset in G^+ sepsis group (n=20).

Figure S4

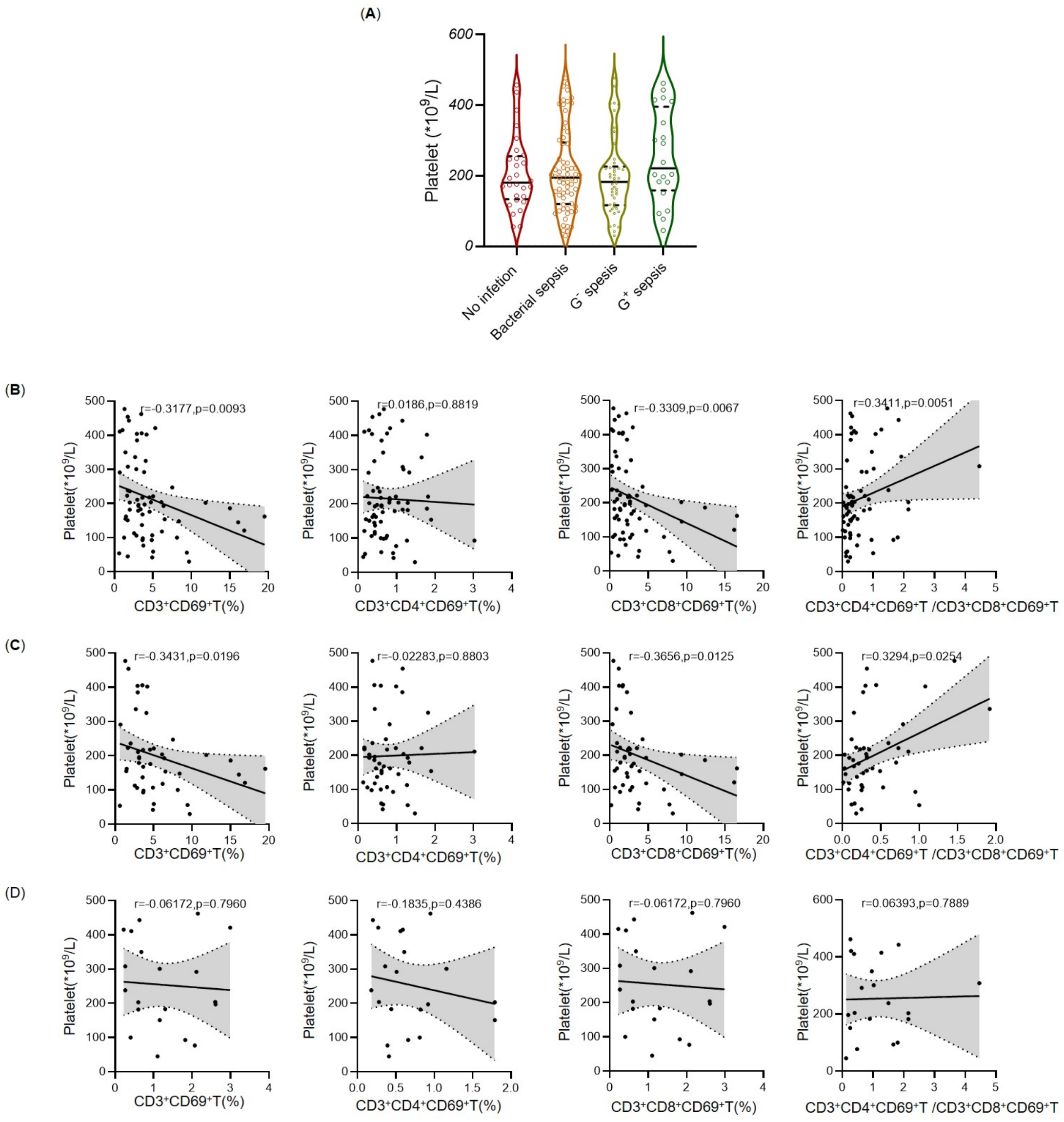

Supplementary Figure S4. The correlations of platelet count with T cell subsets. (A) The comparisons of platelet count in different groups. (B) The correlations of platelet count with T cell subsets in bacterial sepsis group (n=66). (C) The correlations of platelet count with T cell subsets in G^- sepsis group (n=46). (D) The correlations of platelet count with T cell subsets in G^+ sepsis group (n=20).

Table S1. T lymphocyte subpopulation of G^- sepsis group (Pulmonary infection vs. Non-pulmonary infection).

Variables	G^- bacterial sepsis (n=46)	Pulmonary infection (n=24)	Non-pulmonary infection (n=22)	p-Value
WBC (cells/ul)	10730(7070,13662.5)	10605(7157.5,12207.5)	11870(7022.5,15247.5)	0.488
Lymphocyte (cells/ul)	625(425,1020)	535.00 (370.00,995.00)	810.00 (430.00,1247.50)	0.361
T lymphocyte (cells/ul)	416.40(263.36,659.42)	350.01 (261.62,605.19)	484.39 (251.50,881.87)	0.575
B lymphocyte (cells/ul)	93.17(40.95,141.19)	74.89(36.67,140.02)	95.83(43.58,144.60)	0.733
$CD4^+$ T/lymphocyte (%)	36.59±12.97	36.05±11.89	37.18±14.32	0.772
$CD8^+$ T/lymphocyte (%)	26.86(16.17,32.78)	27.37 (17.07,31.08)	24.67 (12.82,34.74)	0.575
$CD4^+$ T/$CD8^+$ T	1.54(0.92, 2.62)	1.52 (0.89,2.25)	1.75 (0.92,2.87)	0.461
NK cell (cells/ul)	116.33(58.67,169.14)	102.80(62.89,128.38)	127.36 (49.31,241.32)	0.339
Regular NK cell (%)	2.98(1.55,5.65)	2.44 (1.22,5.26)	3.85 (2.34,6.97)	0.153
Killer NK cell (%)	96.45(91.98,98.28)	97.35 (94.63,98.77)	95.79 (91.08,97.52)	0.116
Treg /lymphocyte (%)	2.68(1.76,3.97)	2.68 (2.15,3.75)	2.61 (1.143,4.00)	0.613
$CD3^+CD25^+$ T (%)	10.94(6.33,21.42)	11.62 (8.59,21.04)	8.67 (4.02,21.87)	0.156
$CD3^+CD69^+$ T (%)	3.87(2.90,6.41)	3.64 (2.59,4.99)	4.78 (2.92,7.66)	0.416
$CD3^+$HLA-DR^+ T (%)	38.93(29.78,54.34)	40.35 (31.81,54.13)	37.49 (25.78,54.44)	0.328
$CD3^+CD38^+$ T (%)	15.84(8.41,27.14)	17.16 (8.05,26.85)	13.74 (8.44,27.51)	0.668
$CD3^+CD8^+$HLA-DR^+ T (%)	23.26(14.26,34.52)	25.46 (16.21,35.01)	21.51 (10.76,34.52)	0.385
$CD3^+CD4^+CD25^+$ T (%)	8.72(5.73,18.56)	10.86 (7.26,19.46)	7.81 (3.81,18.56)	0.132
$CD3^+CD4^+CD38^+$ T (%)	5.57(3.54,8.34)	5.30 (3.62,7.01)	6.18 (3.49,9.11)	0.567
$CD3^+CD4^+CD69^+$ T (%)	0.60(0.42,0.84)	0.51 (0.41,0.82)	0.61 (0.41,0.93)	0.454
$CD3^+CD4^+$HLA-DR^+ T (%)	12.12(9.05,15.58)	12.12 (10.32,15.49)	11.70 (7.66,15.73)	0.312
$CD3^+CD8^+CD25^+$ T (%)	0.35(0.09,1.08)	0.39 (0.25,1.32)	0.15 (0.08,0.80)	0.088
$CD3^+CD8^+CD38^+$ T (%)	8.66(1.89,16.85)	10.28 (2.39,16.61)	5.33 (1.65,17.14)	0.636
$CD3^+CD8^+CD69^+$ T (%)	2.43(1.30,3.97)	2.26 (1.20,3.07)	2.64 (1.30,5.30)	0.495
$CD3^+CD4^+CD69^+$ T/ $CD3^+CD8^+CD69^+$ T	0.27(0.15, 0.50)	0.26(0.16,0.55)	0.28 (0.14,0.50)	0.904

Data are median (interquartile range) or mean± standard deviation. WBC: white blood cell; NK cell: nature killer cell.

P-Value for the comparison between Pulmonary infection group and Non-pulmonary infection group; p-Values were estimated by Mann–Whitney U test or t test.

Table S2. T lymphocyte subpopulation of bacterial sepsis group (Pulmonary infection vs. Non-pulmonary infection).

**Variables**	**bacterial sepsis (n=66)**	**Pulmonary infection (n=27)**	**Non-pulmonary infection (n=39)**	**p-Value**
WBC (cells/ul)	10975(7727.5,14132.5)	10290 (6600,12050)	12810 (8060,15330)	0.091
Lymphocyte (cells/ul)	755(430,1132.5)	530 (360,980)	830 (440,1390)	0.107
T lymphocyte (cells/ul)	432.71(256.30,721.11)	340.40 (258.85,595.03)	495.26 (237.01,942.43)	0.251
B lymphocyte (cells/ul)	95.82(48.89,238.68)	75.48 (38.36,139.46)	108.29 (50.43,255.81)	0.206
$CD4^+$ T/lymphocyte (%)	37.78±12.50	36.48±12.05	38.68±12.88	0.481
$CD8^+$ T/lymphocyte (%)	20.93(13.79,30.66)	24.82 (16.77,30.07)	18.32 (11.81,31.53)	0.088
$CD4^+$ T/$CD8^+$ T	2.00(1.02,3.10)	1.57 (0.96,2.25)	2.15 (1.20,3.59)	0.088
NK cell (cells/ul)	112.01(58.29,166.22)	97.80 (60.46,128.74)	121.40 (51.30,175.05)	0.602
Regular NK cell (%)	3.43(1.55,6.42)	2.7 (1.24,6.17)	3.54 (1.63,6.69)	0.445
Killer NK cell (%)	95.79(91.16,98.28)	97.1 (93.48,98.72)	95.41 (90.98,97.63)	0.206
Treg /lymphocyte (%)	2.77(2.16,3.97)	2.67 (2.07,3.28)	2.82 (2.17,3.97)	0.553
$CD3^+CD25^+$ T (%)	11.34(6.71,21.42)	11.44 (8.46,19.96)	10.54 (5.9,22.53)	0.540
$CD3^+CD69^+$ T (%)	3.60(1.96,5.04)	3.63 (2,5.02)	3.54 (1.88,5.10)	0.759
$CD3^+$HLA-DR^+ T (%)	37.21(27.53,53.65)	39.83 (31.61,55.39)	32.42 (22.51,53.53)	0.062
$CD3^+CD38^+$ T (%)	13.74(8.06,24.07)	16.64 (7.93,27.08)	12.04 (8.1,23.08)	0.348
$CD3^+CD8^+$HLA-DR^+ T (%)	20.02(11.59,32.54)	25.32 (16.12,33.09)	14.28 (8.21,32.36)	**0.036**
$CD3^+CD4^+CD25^+$ T (%)	9.85(6.28,18.34)	10.80 (7.74,16.98)	8.96 (5.28,18.95)	0.361
$CD3^+CD4^+CD38^+$ T (%)	5.46(3.54,8.34)	5.17 (3.54,6.62)	5.76 (3.52,9.86)	0.379
$CD3^+CD4^+CD69^+$ T (%)	0.61(0.42,1.01)	0.59 (0.42,0.82)	0.66 (0.39,1.16)	0.334
$CD3^+CD4^+$HLA-DR^+ T (%)	12.95(9.21,15.58)	12.98 (11.28,16.34)	12.92 (8.69,15.43)	0.270
$CD3^+CD8^+CD25^+$ T (%)	0.38(0.13,0.99)	0.38 (0.26,1.31)	0.38 (0.1,0.94)	0.411
$CD3^+CD8^+CD38^+$ T (%)	4.47(1.91,13.73)	10.03 (1.92,16.65)	3.36 (1.86,12.23)	0.179
$CD3^+CD8^+CD69^+$ T (%)	2.00(0.94,3.02)	2.22 (0.95,2.99)	1.93 (0.63,3.53)	0.411
$CD3^+CD4^+CD69^+$ T/	0.32(0.19,0.87)	0.28 (0.18,0.70)	0.38 (0.19,1.00)	0.288

CD3$^+$CD8$^+$CD69$^+$ T

Data are median (interquartile range) or mean± standard deviation. WBC: white blood cell; NK cell: nature killer cell.

P-Value for the comparison between Pulmonary infection group and Non-pulmonary infection group; p-Values were estimated by Mann–Whitney U test or t test.

Table S3 Univariate and multivariate logistic regression analyses of G$^-$ sepsis (versus G$^+$ sepsis)

Variables	Univariate logistic regression		Multivariate logistic regression	
	OR (95% CI)	P.value	OR (95% CI)	P.value
CD3+CD69+ T (%)	1.562(1.100,2.217)	**0.013**		
CD3$^+$CD8$^+$CD69$^+$T(%)	2.169(1.217,3.865)	**0.009**		
CD3$^+$CD8$^+$HLA-DR$^+$ T (%)	1.058(1.008,1.111)	**0.022**		
CD4$^+$ T/CD8$^+$ T	0.614(0.413,0.913)	**0.016**		
CD3$^+$CD4$^+$CD69$^+$ T / CD3$^+$CD8$^+$CD69$^+$ T	0.157(0.051,0.482)	**0.001**	0.078(0.012,0.506)	**0.008**
CD3$^+$CD8$^+$T (%)	1.089(1.021,1.161)	**0.01**		
PCT(＞0.53 vs ≤0.53ng/ml)	6.167(1.945,19.547)	**0.002**	9.31(1.36,63.58)	**0.023**
CO2CP (＜26.5 vs ≥26.5 mmol/L)	3.463(1.153,10.399)	**0.027**	10.99(1.29,93.36)	**0.028**
Infection sites (vs Other infections)				0.061
Pulmonary infection	6.182(1.592,24.011)	**0.009**		
Bloodstream infection	0.257(0.080,0.825)	**0.022**		
Thoracic or abdominal infection	1.615(0.305,8.562)	0.573		
Wound or soft tissue infection	0.209（0.045,0.983）	0.209		
Age (10-year increments)	1.064（0.742,1.526）	0.736		
Sex (male vs female)	2.794（0.947,8.242）	0.063		

OR, odd ratio; 95%CI, 95% confidence interval; CO_2CP, carbon dioxide combining power; PCT, procalcitonin.

Table S4. Cox regression analysis of factors associated with the hospital discharge probability for G^- septic patients.

Variables	Category	Univariabale cox regression			Multivariable cox regression		
		HR	95%CI	P.value	HR	95%CI	P.value
Age	10-year increments	0.97	0.8-1.18	0.788			
B lymphocyte (cells/ul)	≥93.17 vs <93.17	1.83	0.95-3.5	0.069			
$CD3^+CD8^+CD69^+$T(%)	≥2.430 vs <2.430	0.44	0.23-0.85	**0.014**	0.59	0.28-1.24	0.166
$CD3^+CD8^+CD38^+$T(%)	≥8.66 vs <8.66	0.42	0.21-0.81	**0.01**	0.54	0.25-1.15	0.11
$CD3^+CD8^+$HLA-DR^+T(%)	>23.255 vs ≤23.255	0.66	0.35-1.24	0.196			
Abs for GPB	yes vs no	0.62	0.33-1.16	0.136			
IL-6 (pg/ml)	≥60.19 vs<60.19	0.59	0.32-1.1	0.095			

HR, hazard ratio; 95%CI, 95% confidence interval; IL, interleukin; Abs, antibiotics; GPB: gram positive bacteria.
